# Correlation between structural heterogeneity and plastic deformation for phase separating FeCu metallic glasses

**DOI:** 10.1038/srep34340

**Published:** 2016-09-29

**Authors:** Chuan-Xiao Peng, Kai-Kai Song, Li Wang, Daniel Şopu, Simon Pauly, Jürgen Eckert

**Affiliations:** 1 School of Mechanical, Electrical & Information Engineering, Shandong University (Weihai), WenhuaXilu 180, 264209 Weihai, P.R. China; 2 IFW Dresden, Institute for Complex Materials, Helmholtzstraße 20, D-01069 Dresden, Germany; 3 ErichSchmid Institute of Materials Science, Austrian Academy of Sciences, Jahnstraße 12, A-8700 Leoben, Austria; 4 Department Materials Physics, Montanuniversität Leoben, Jahnstraße 12, A-8700 Leoben, Austria

## Abstract

Unlike crystalline metals, the plastic deformation of metallic glasses (MGs) involves a competition between disordering and structural relaxation ordering, which is not well understood, yet. Molecular dynamics (MD) simulations were performed to investigate the evolutions of strain localizations, short-range order (SRO) as well as the free volume in the glass during compressive deformation of Fe_50_Cu_50_ MGs with different degrees of phase separation. Our findings indicate that the free volume in the phase separating MGs decreases while the shear strain localizations increase with increasing degree of phase separation. Cu-centered clusters show higher potential energies and Voronoi volumes, and bear larger local shear strains. On the other hand, Fe-centered pentagon-rich clusters in Cu-rich regions seem to play an important role to resist the shear transformation. The dilatation or annihilation of Voronoi volumes is due to the competition between ordering via structural relaxation and shear stress-induced deformation. The present study could provide a better understanding of the relationship between the structural inhomogeneity and the deformation of MGs.

In recent years, a fascinating class of metallic materials called metallic glasses (MGs) with intriguing mechanical properties especially with respect to their unique room temperature mechanical properties has been extensively and in-depth studied by researchers^1^,^2^,^3^. At room temperature, the plastic deformation of MGs is localized within shear bands, being different from their crystalline counterparts, which causes brittleness and catastrophic failure during deformation^4^,^5^. This severely hampers the practical applications of MGs as structural materials. Recently, various methods have been adopted to enhance the room-temperature ductility/toughness of MGs by creating a large amount of free volume in MGs^6^,^7^,^8^, introducing atomic-scale or nanocrystalline inhomogeneity^9^,^10^, enhancing the amount icosahedral short-range order (ISRO) and favoring ISRO-mediated local distribution^11^, and introducing nanoscale phase separation into MGs^12^, respectively. As a result, the fabricated MGs based on such strategies indeed show improved mechanical properties at room temperature. However, the correlation between different structures induced by composition variation and different processing histories and their mechanical properties is still not very clear.

Therefore, a major challenge is to clarify structural differences in these seemingly structureless alloys and to establish a causal link between key local structures and macroscopic mechanical properties^13^,^14^,^15^. Recently, molecular dynamics (MD) simulations have been widely used to explore the structure-mechanical property relationship by describing the general features of local structures and their evolutions associated with shear deformation^2^,^16^,^17^. In order to establish such a relationship, more and more attention has been paid to atomic-level features, i.e. short-range order (SRO) or medium-range order (MRO) of structures^14^,^15^,^16^,^17^,^18^,^19^,^20^,^21^, free volume^18^,^21^,^22^, and atomic-level stresses^14^,^23^. It has been reported that the stability of pentagon-rich clusters in the glassy state especially for full icosahedra plays a key role in slowing down the dynamics^24^, forming a more extended and stronger elastic backbone resisting local shear deformation^15^,^17^,^18^,^25^,^26^, or showing lower strain energies^14^. Shear transformation in MGs preferentially occurs at a localized soft phase, and geometrically unfavored motifs constitute the most flexible local environments that encourage soft modes and high propensity for shear transformations^27^. Free volume increases preferentially within strain localization regions or shear bands upon compression^18^, tension^21^, or shearing^22^ of samples.

Until now, most studies have focused on Cu-Zr-(Al)^2^,^13^,^16^ MGs. However, phase separating MGs which have typical heterogeneous characteristics are rarely involved, and thus the corresponding shear localizations and free volume distributions in phase separating MGs during deformation are not well understood. In this paper, we present a MD study on the compressive deformation of phase separating Fe_50_Cu_50_ MGs at a temperature of 100 K in an attempt to clarify the structural evolutions relating to shear strain localizations at an atomic level.

## Results and Discussion

### Stress-strain curve and strain localization

**Figure 1:**
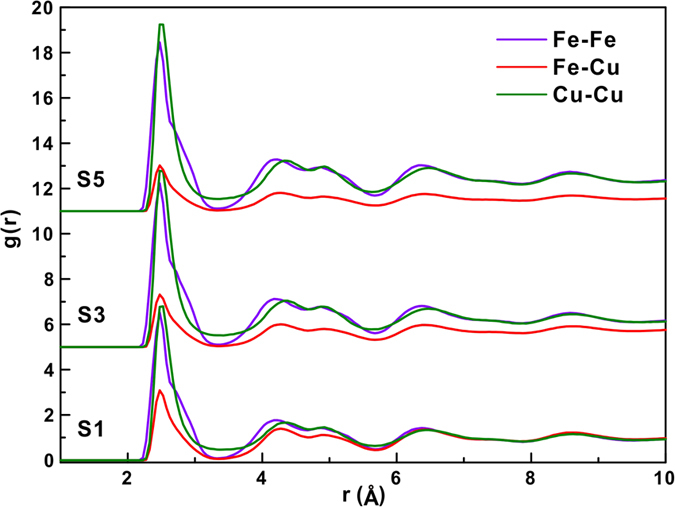
Pair correlation functions of S1, S3 and S5.

**Figure 2:**
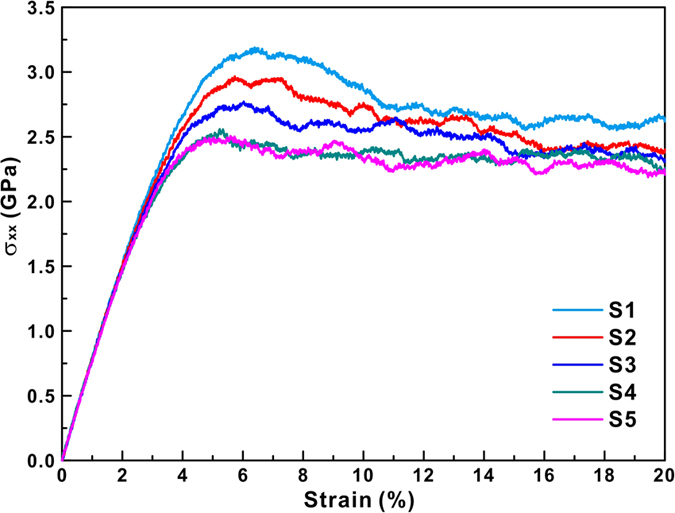
Stress-strain curves of S1–S5 samples.

Figure 1 shows the pair correlation functions (PCFs)^2^ of the S1, S3 and S5 samples first relaxed before compression. Stronger interactions between homogeneous atom pairs (i.e. Fe-Fe and Cu-Cu, respectively) and weaker interactions between heterogeneous atom pairs (i.e. Fe-Cu) can be observed with increasing degree of phase separation in Fe_50_Cu_50_ MGs. A splitting of the second peak in the PCF curve is observed, which is usually regarded as a characteristic indication for glass formation. However, recent reports have shown that the splitting of the second peak is due to a statistical average of crystal-like and disordered structural regions in the glass^28^. In addition, we calculated the nearest neighbor correlations index C_*ij*_ to quantify the degree of phase segregation, the value of C_*FeFe*_ and C_*CuCu*_ increase while the value of C_*FeCu*_ decrease after a longer relax time, it implied that the degree of phase segregation should be enhanced. The stress-strain curves for MGs with different degree of phase separation (Fig. 2) present the same initial slope during elastic deformation. The elastic regime of the S1 and S2 samples corresponds to a strain of about 4%, while the S3, S4 and S5 samples show a lower elastic strain below about 3%. The yield points decrease with increasing degree of phase separation in the MGs. It seems that the more phase separated samples have a lower elastic limit and are softer due to the more pronounced phase separation introduces some more “soft spots” and more “fertile zones” for shear transformations. Beyond yielding, the corresponding stress does not drop sharply. Previous reports have demonstrated that the sharp drop of the stresses after yielding can be related to a single shear banding process^18^, which in turn implies that the samples in our case could show multiple shear location states during deformation. Furthermore, as shown in Fig. 2, all simulated samples display a high plastic flow, not a sudden and rapid drop in stress.

**Figure 3:**
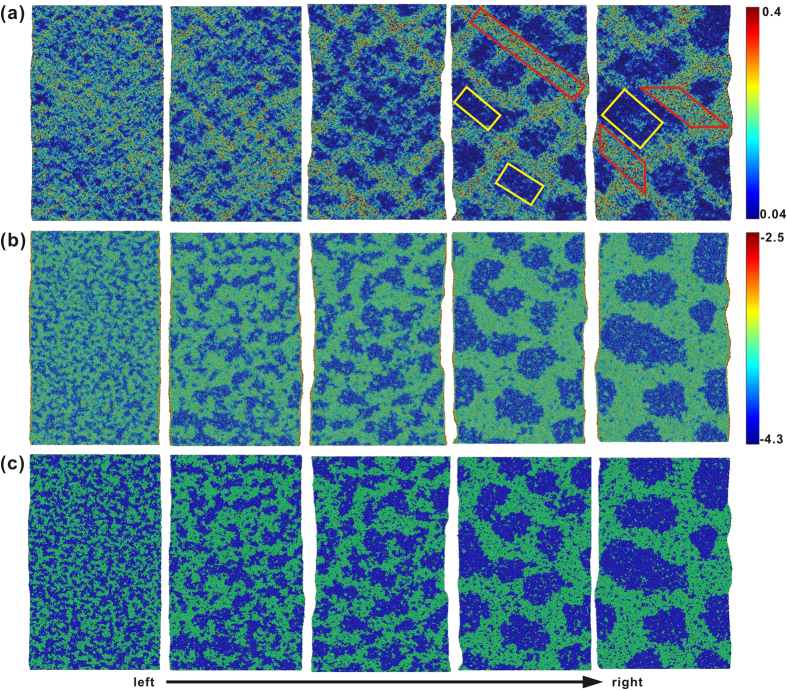
Atomic configurations of S1–S5 (from left to right) at a strain of 12%. (**a**) Atoms are colored according to the local shear strain 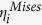. In S4 and S5, atoms in strain localization regions and matrix are selected for further analysis. (**b**) According to the atomic potential energies in units of eV. (**c**) According to atomic type, blue and green represent Fe and Cu atoms, respectively.

The deformation process is monitored by the atomic local shear strains measured by 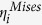. As a comparison, we chose the atomic configurations of the S1, S2, S3, S4 and S5 samples at a strain of 12% and take 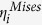 as a color ruler (Fig. 3(a)). Relatively large local shear strains develop not only on the free surface but even in the early elastic regime. The distribution of the local shear strains changes from homogeneous to heterogeneous with increasing degree of phase separation in the MGs. Comparing the local shear strains with the atomic type configurations in Fig. 3(c) reveals that larger shear strains localize in Cu-rich regions and distribute more homogeneously in the S1 and S2 samples, which is correlated with the initial structural heterogeneity^2^. The shear offsets grow at the sides of the samples where a larger shear strain propagates across the entire sample. Although the potential energies may ambiguously partition the atomic-level stresses^14^, they are often used to represent the changes of the local structure characteristics^18^,^20^,^22^. As shown in Fig. 3(b), the Cu-rich regions show higher energies than Fe-rich regions, implying that inherently high energies could provide a drive force during deformation. Therefore, for Fe_50_Cu_50_ MGs, Cu-rich regions can be treated as soft or liquid-like regions compared with Fe-rich regions.

**Figure 4:**
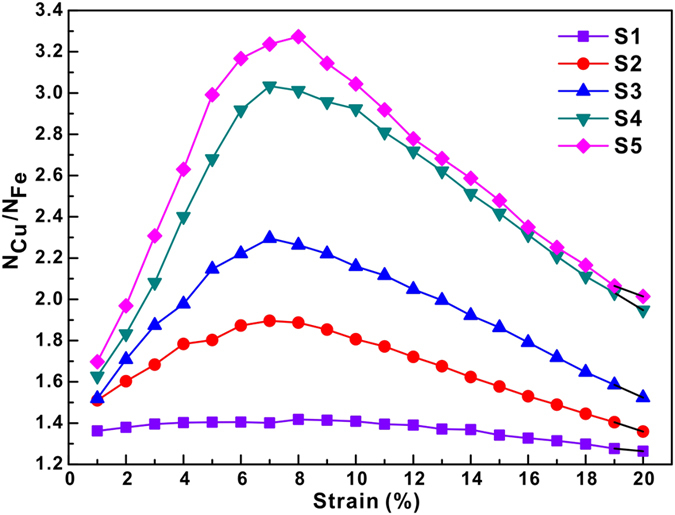
Ratio of atomic number *N*_*Cu*_*/N*_*Fe*_ changing with the sample strain, where *N*_*Cu*_ and *N*_*Fe*_ are the atom numbers of Cu and Fe whose local shear strain is larger than the average local shear strain, respectively.

Figure 4 shows the ratios of the atom number *N*_*Cu*_*/N*_*Fe*_, where *N*_*Cu*_ and *N*_*Fe*_ are the atom numbers of Cu and Fe, respectively, whose local shear strain is larger than the average local shear strain. The average local shear strain is calculated based on the following relationship: 
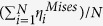
, where *N* is the total atom number. The *N*_*Cu*_*/N*_*Fe*_ ratio is larger than 1.0, implying that more Cu atoms have undergone a larger local shear strain than Fe atoms. Even for the S1 sample, the corresponding *N*_*Cu*_*/N*_*Fe*_ ratio is almost larger than 1.3 throughout the whole deformation process. Besides, the *N*_*Cu*_*/N*_*Fe*_ ratio increases when the degree of phase separation increases in the MGs, which corresponds well with the large shear strains localizing in the Cu-rich regions mentioned above. Furthermore, the *N*_*Cu*_*/N*_*Fe*_ ratio increases first and then decreases again with increasing strain, with a maximum locating at a strain of 7–8%. This means that the shear strain localization firstly propagates along the Cu-rich regions and then extends to the neighboring Fe-rich regions. Hence, these findings imply that the shear band may broaden by incorporating more and more material from the neighboring matrix^29^. The volume fraction of atoms which undergo a larger local shear strain than the average value over the whole sample (termed LLSS volume fraction hereafter) was also calculated by (*N*_*Cu*_ + *N*_*Fe*_)*/N*, thus being quite similar to the parameters of the deformation participation ratios (DPR)^30^. In a homogeneously deforming sample, the LLSS volume fraction should be approximately 0.5 and the LLSS volume fraction should decrease due to localized deformation^30^. For all the phase separating MGs, the LLSS volume fractions decrease first and then increase during deformation. However, during deformation, the LLSS volume fractions for the sample with higher degrees of phase separation are always smaller than those for ones with lower degrees of phase separation. The minimum LLSS volume fraction with changing strains is still as high as 0.358, implying the occurrence of relatively homogeneous deformation. However, the transition from homogenous to heterogeneous deformation can be still clearly observed.

### SRO and Voronoi volume in clusters

In order to achieve an atomistic level description of the deformation mechanism of different phase separating Fe_50_Cu_50_ MGs, we have analyzed the overall as well as local changes in topological SRO characterized by the Voronoi index (*n*_*3*_, *n*_*4*_, *n*_*5*_, *n*_*6*_), the chemical SRO calculated by the nearest neighbor correlation index *C*_*ij*_, and the Voronoi volume during deformation, respectively. It should be noted that the standard approach has been used to prescribe free boundary conditions when facing with an irregular cluster of particles. As a result, the Voronoi cells for particles at the edges of the packing would be very large and then extend off to the edges of the calculation box. For example, the Voronoi cells were found to be as large as 47 faces in MG nanowires^31^. In order to accurately calculate Voronoi cells for the entire samples, the very thin atomic layer at the free surfaces was carefully removed. This way selected entire sample contains still more than 96% of the total atoms, which should not affect the calculated accuracy. Meanwhile, the criterion for tiny surfaces was also found to affect the Voronoi index and thus the tiny surface whose area is less than 0.5 Å^2^ was removed in the present simulations.

**Figure 5:**
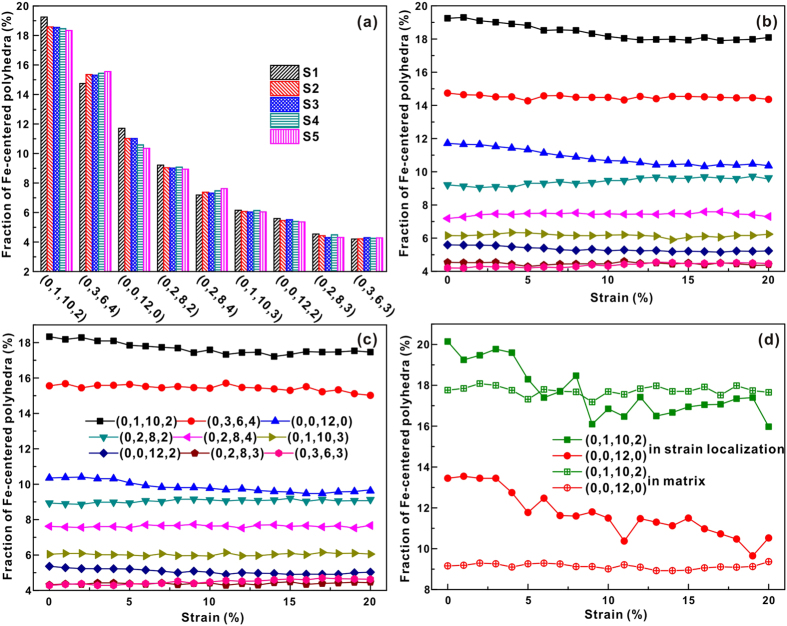
(**a**) Distribution of major Fe-centered Voronoi polyhedra of the entire S1–S5 samples before loading. Fraction of Fe-centered Voronoi polyhedra of (**b**) S1, (**c**) S5, and (**d**) S5 in different regions as a function of sample strain.

**Figure 6:**
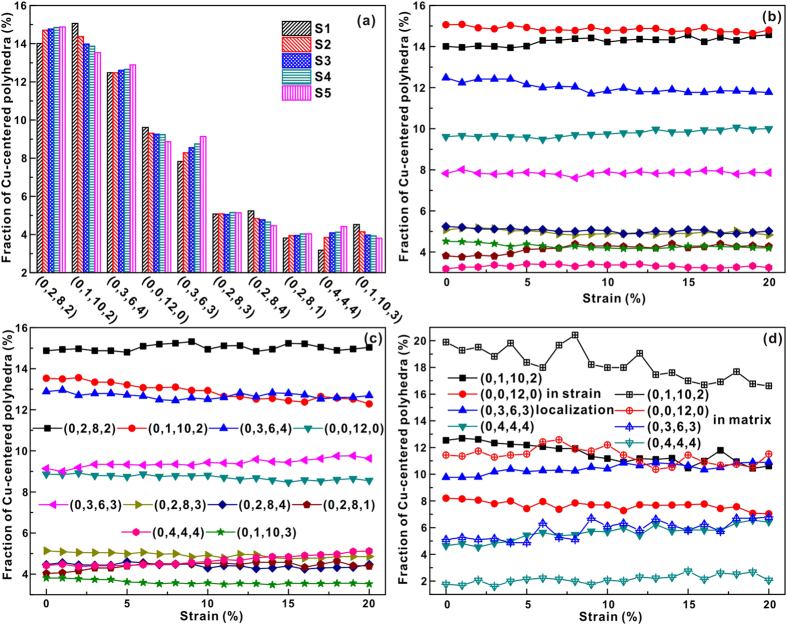
(**a**) Distribution of major Cu-centered Voronoi polyhedra of the entire S1–S5 samples before loading. Fraction of Cu-centered Voronoi polyhedra of (**b**) S1, (**c**) S5, and (**d**) S5 in different regions as a function of sample strain.

The distributions of Fe-centered and Cu-centered Voronoi polyhedra (>4%) for all the computed samples before compression are shown in Figs 5(a) and Fig. 6(a), respectively. The polyhedra indexed by (0, 1, 10, 2) and (0, 3, 6, 4) are popular in Fe-centered clusters, while Cu-centered clusters are governed by the index (0, 2, 8, 2), (0, 1, 10, 2), and (0, 3, 6, 4) polyhedra. In our case, the index (0, 0, 12, 0) polyhedra, which were found to be the predominant structural motif in Cu_64_Zr_36_ MGs^15^,^18^,^20^,^21^, are less both Fe-centered and Cu-centered polyhedra, respectively. The numbers of (0, 1, 10, 2) and (0, 0, 12, 0) polyhedral in Fe-centered clusters show a slight decrease with increasing degree of phase separation in MGs, while the (0, 3, 6, 4) and (0, 2, 8, 4) polyhedral exhibit an opposite tendency. The numbers of Cu-centered clusters change more than those of Fe-centered ones when the degree of phase separation in MGs increases. The numbers of (0, 1, 10, 2), (0, 0, 12, 0), (0, 2, 8, 4), and (0, 1, 10, 3) polyhedral in Cu-centered clusters decrease in the MGs, while those of (0, 2, 8, 2), (0, 3, 6, 4), (0, 3, 6, 3), and (0, 4, 4, 4) increase with increasing degree of phase separation. Especially for Cu-centered clusters the structure seems to become more ordered with increasing degree of phase separation. Since all samples exhibit a similar deformation tendency, the S1 and S5 samples were chosen to investigate the cluster evolutions during deformation in more detail (Figs 5(b,c) and 6(b,c)). Atoms in strain localized regions and in the matrix were selected in the S4 and S5 samples (the regions marked by the boxes in Fig. 3(a)) for further analysis. Obviously, more Cu atoms locate within the strain localization regions while more Fe atoms are distributed in the matrix. Therefore, it is reasonable to treat the strain localizations as Cu-rich regions and the matrix as Fe-rich regions, respectively. Figures 5(d) and 6(d) display the Fe-centered and Cu-centered Voronoi polyhedral in different regions of the S5 MG. In the following, the S5 samples were chosen to analyze the changes of the topological and chemical SROs, as well as the free volume in strain localized regions and the matrix with increasing degree of deformation. During deformation, the numbers of pentagon-rich clusters (0, 1, 10, 2) and (0, 0, 12, 0) decrease in Fe-centered polyhedra, while others are almost unchanged. It can also be deduced from Fig. 5(d) that the decrease in the number of Fe-centered polyhedral mainly comes from the strain localization regions. The numbers of Fe-centered (0, 1, 10, 2) and (0, 0, 12, 0) clusters decrease when the strain is larger than about 3% within the strain localization regions, while they remain almost unchanged in matrix for the whole deformation process. Full icosahedra often show lower strain energies and potential energies, which could slow down the dynamics or resist local shear transformation by forming a network-like connection^14^,^15^,^17^,^18^. In our case, although the numbers of full icosahedra do not drop dramatically during the formation of shear bands^18^, the numbers of Fe-centered pentagon-rich clusters continuously decrease, indicating that Fe-centered pentagon-rich clusters should play a key role in resisting shear transformation.

For Cu-centered polyhedra, even though the numbers of the (0, 2, 8, 1) clusters in all the investigated samples increase during deformation, their content is very low. In the S4 and S5 samples, the numbers of the (0, 1, 10, 2) and (0, 0, 12, 0) clusters decrease and those of (0, 3, 6, 3) and (0, 4, 4, 4) clusters increase with increasing strain. On the contrary, such changes were not found in the S1, S2 and S3 samples. These observations may be attributed to a decrease of the Fe content around Cu-centered clusters under the shear stress. Since more homogeneous atoms gather together with increasing degree of phase separation, the contents of Fe around Cu-centered clusters (even in Fe-rich regions) in the S4 and S5 samples are lower than those in the S1, S2 and S3 samples. This further confirms the supportive role of Fe atoms in the samples during deformation. As shown in Fig. 6(d), the numbers of the Cu-centered (0, 1, 10, 2) and (0, 0, 12, 0) clusters decrease both in strain localization regions and the matrix in the S5 samples, while the numbers of (0, 4, 4, 4) and (0, 3, 6, 3) clusters decrease within the strain localization regions. The Cu-centered polyhedra also seem to become more ordered with increasing strain, especially in strain localization regions.

**Figure 7:**
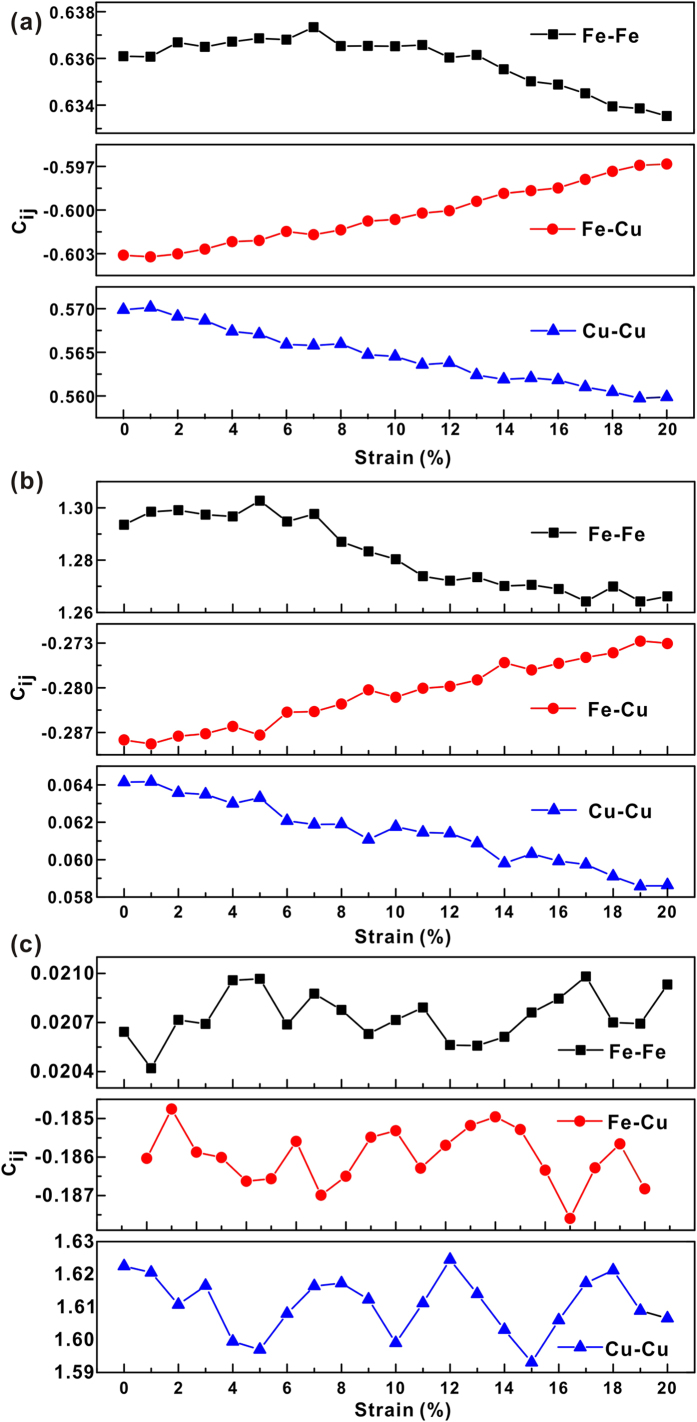
The nearest neighbor correlation index *C*_*ij*_ in (**a**) entire sample, (**b**) strain localization region and (**c**) matrix of S5 as a function of sample strain.

In order to check the changes of the chemical SRO in the S5 samples, the nearest neighbor correlations indexed *C*_*ij*_ in the strain localization regions and the matrix were calculated, respectively, and are shown in Fig. 7. For the entire sample,*C*_*FeFe*_ and *C*_*CuCu*_ decrease, while *C*_*FeCu*_ increases during plastic deformation. Within strain localization regions, a decrease of *C*_*FeFe*_, an increase of *C*_*FeCu*_ at strains larger than about 5%, and a successive decrease of *C*_*CuCu*_ can be observed. Meanwhile, the numbers of the Fe-Fe and Cu-Cu bonds decrease, while the numbers of Fe-Cu bonds increase during plastic deformation. In the matrix, the *C*_*FeFe*_, *C*_*FeCu*_, and *C*_*CuCu*_ fluctuate within a certain range without monotonous increase or decrease, indicating that the bonds are more stable than those in the strain localization regions.

**Figure 8:**
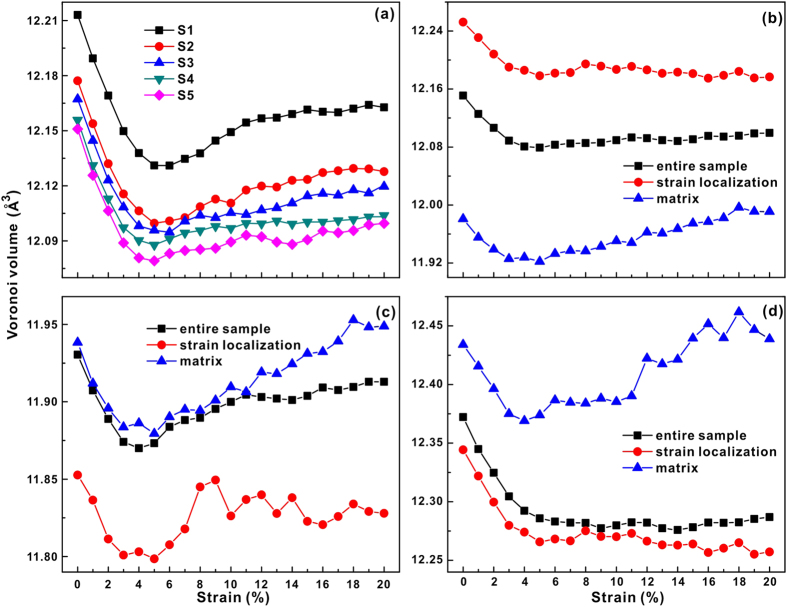
Voronoi volume as a function of sample strain. (**a**) Average per atom Voronoi volume of the entire S1–S5 samples. (**b**) Average per atom Voronoi volume, (**c**) Atom Fe Voronoi volume and (**d**) Atom Cu Voronoi volume of S5 in different regions.

**Figure 9:**
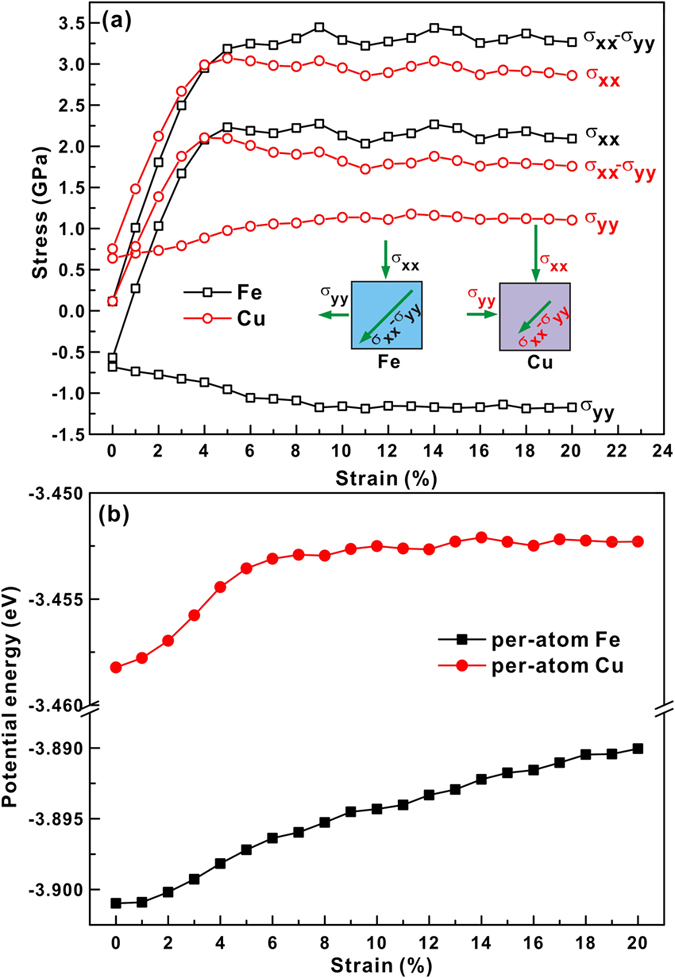
(**a**) Stress state and (**b**) Potential energies of Fe and Cu in the entire S5 sample as a function of sample strain.

Such observations could imply that the changes in the chemical SRO may be closely related to changes in free volume. The strain dependence of the Voronoi volume is shown in Fig. 8. The average per atom (APA) Voronoi volumes (i.e. the total volumes divided by the total numbers of atoms in selected regions) for the S1, S2, S3, S4 and S5 samples are shown in Fig. 8(a). Apparently, the APA Voronoi volume decreases with increasing degree of phase separation. The samples with larger APA Voronoi volumes (i.e. less densely packed) seem to be deformed more homogenously. This also supports the view that the more densely packed the material is relative to the flowing steady state, the more likely the system is to experience instability, resulting in the localization of shear banding^32^. The APA Voronoi volume is compressed during elastic deformation and is recovered during plastic deformation, a feature that was also found in Feng *et al*.’s work^33^. The Voronoi volume per Cu atom is about 12.25–12.45 Å^3^, being larger than that of Fe of 11.8–11.95 Å^3^ (Figs. 8(c–d)). Therefore, as shown in Fig. 8(b), the APA Voronoi volumes within the strain localization regions (i.e. Cu-rich regions) are larger than those in the matrix (i.e. Fe-rich regions). Interestingly, during plastic deformation, the APA Voronoi volumes slightly increase and then slightly drop again at a strain of about 8% in strain localization regions, but continuously grow in the matrix (Fig. 8(b)). The change of free volume in MGs during plastic deformation involves two competing events, i.e. ordering via structural relaxation-induced annihilation and shear-induced dilatation^34^,^35^,^36^. The clusters in the strain localization regions (i.e. Cu-centered regions) rearrange to become more ordered during plastic deformation while free volume may be annihilated. Here, the plane stresses (i.e. *σ*_*xx*_, *σ*_*yy*_ and *τ*_*max*_ = *σ*_*xx*_ − *σ*_*yy*_) of Fe and Cu in the whole S5 sample were calculated, as shown in Fig. 9(a). Positive principal stresses *σ*_*xx*_ and *σ*_*yy*_ represent compressive stresses at different directions. The calculations reveal different stress states of Fe and Cu during compression. At the beginning of compression, Fe atoms bear a tensile stress (i.e. negative *σ*_*xx*_ and *σ*_*yy*_) and Cu atoms bear a compressive stress (i.e. positive *σ*_*xx*_ and *σ*_*yy*_), while the shear stress *τ*_*max*_ on both Fe and Cu is almost at zero level. During plastic deformation, Fe atoms suffer a tensile transverse stress σ_yy_ and show a larger τ_max_ than Cu atoms. The τ_max_ of Cu atoms slightly drops during plastic deformation, which indicates a release of the shear stress acting on Cu atoms to some degree. Furthermore, the average potential energies per atom for Fe and Cu at different strains were also calculated, respectively, which is shown in Fig. 9(b). It can be seen that the average potential energy per for Fe atom continuously grows while Cu achieves a balance after elastic deformation. The Fe-rich matrix continuously elastically extend under the applied stress resulting in a increase in the average interatomic distance and consequently, to a linear grows of the average potential energy. Besides, as also revealed by the evolution in the SRO and Voronoi volume under the applied strain, the level of *interatomic distance* in Cu-rich regions does not considerable change after the yield point leading to a near constant potential energy evolution during deformation.

The Fe atoms can sustain a larger shear stress, which further validates the backbone function of Fe atoms in the samples. Besides, the stress states in matrix (Fe-rich) and strain localization zones (Cu-rich) should be different due to the different stress states of Fe atoms and Cu atoms, which should induce a different tendency for the change of the Voronoi volume in matrix and strain localization regions, respectively. Another point is that the atomic arrangement, i.e. the topological and chemical SRO, changes more intensively in strain localization regions than in the matrix. The accumulation of the shear stress τ_max_ and the rather slow structural relaxation lead to the dilatation of free volume in matrix. However, in case of strain localization regions, the Voronoi volume increases slightly during straining up to 5–8%, and then drops slightly at a strain of 8% during plastic deformation. This also can be correlated with the competition between the ordering via structural relaxation-induced annihilation of free volume and shear-induced dilatation^34^,^35^,^36^. The chemical SRO of Fe-Cu increases linearly with strain localization (Fig. 7(b)), meaning that Fe atoms move towards Cu atoms. As Fe or Cu atoms are removed, other atoms have not enough time to occupy the empty position left by the moved atoms, possibly leading to the slight increase of the Voronoi volume at strains of 5–8%. Then the APA Voronoi volume remains almost unchanged since the competition between ordering via structural relaxation-induced free volume annihilation and shear-induced dilatation achieves a balance during deformation.

**Figure 10:**
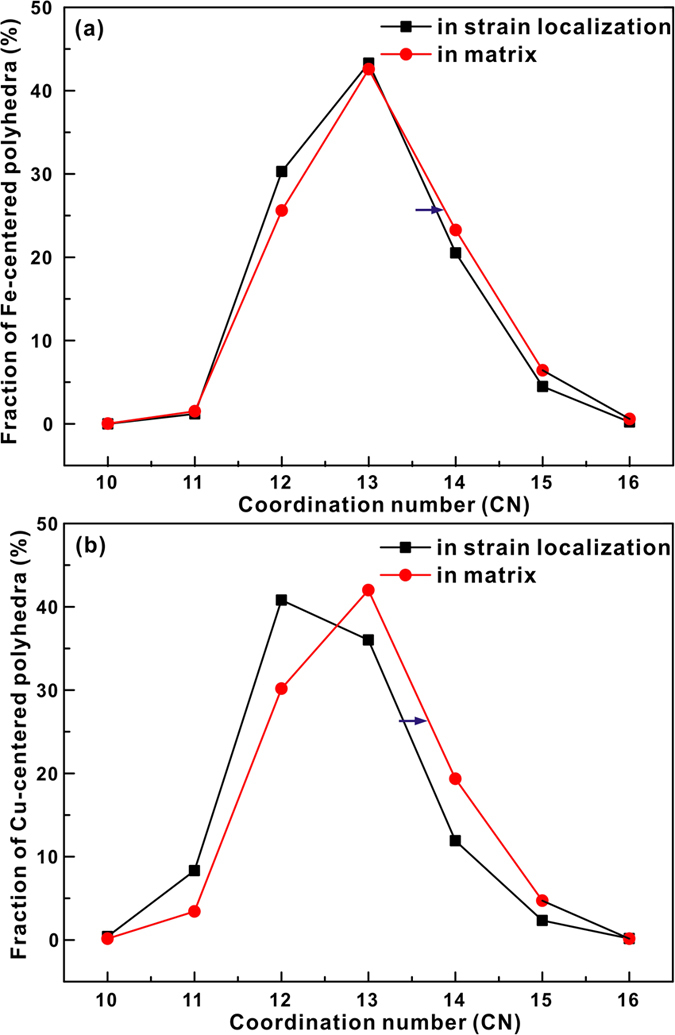
Distribution of (**a**) Fe-centered and (**b**) Cu-centered Voronoi polyhedra of S5 before deformation sorted by coordination number.

The individual Voronoi volumes of Fe and Cu (Fe or Cu volume divided by the number of Fe or Cu atoms in the selected region, respectively) were also calculated (Fig. 8(c–d)). The individual Voronoi volume is smaller in the strain localization regions than in the matrix for both Fe atoms and Cu atoms. For Fe atoms in strain localization regions, the excess volume is relatively larger due to the smaller individual Voronoi volume and the larger APA Voronoi volume in strain localization regions than in the matrix. For the entire sample, the Voronoi volume of Fe dilates while that of Cu remains almost unchanged during plastic deformation, which indicates that Cu atoms preferentially undergo flow deformation. This is also manifested by the stress states and potential energies as a function of strain (Fig. 9). The shear stress sustained by Fe atoms is at a quite larger level and their corresponding potential energies always increase during deformation. Meanwhile, the shear stress sustained by Cu atoms is released to some degree and their potential energies remain unchanged. Although the Voronoi volume cannot fully reflect the complex relationship between structural relaxation and shear stress state, its dilatation or annihilation can be treated as the results of the competition between structure relaxation ordering and shear stress-induced deformation. In addition, the Voronoi volume is inherently smaller in strain localization regions than in the matrix, which could be caused by the structural heterogeneity of the present MGs as well as different stress state. Figure 10 depicts the correlation between the distributions of the Fe-centered and Cu-centered Voronoi polyhedral in the S5 sample and their coordination numbers before deformation. Both the Fe-centered and the Cu-centered clusters possess a larger coordination number in the matrix than in the strain localization regions. It can be concluded that for these seemingly structureless atoms, a larger coordination number packing generates statistically a larger Voronoi volume.

## Conclusions

In summary, we have performed classical molecular dynamics simulations to study the deformation processes of phase separated Fe_50_Cu_50_ MGs with different degree of separation upon compression. From the investigations we can draw the following conclusions: (1) With increasing degree of phase separation, the samples become more heterogeneous, and Cu-rich regions and Fe-rich regions can be observed more obviously. Although the Voronoi volume becomes smaller with increasing degree of phase separation, structural ordering is enhanced through the decrease of the number of pentagon-rich clusters. (2) Cu-centered clusters show high potential energies, Voronoi volume and localize larger atomic shear strains. The number of Fe-centered pentagon-rich clusters decreases gradually during deformation and plays a role in resisting shear transformation in our Fe-Cu MG simulations. It is further validated by the stress state that Fe atoms bear larger shear stresses than Cu atoms and the shear stress on Cu atoms is released to some degree during plastic deformation. (3) The Voronoi volume of Cu is bigger than that of Fe, and the Voronoi volume of Fe or Cu in the matrix is larger than in strain localization regions due to the larger coordination number packing in the matrix. Fe and Cu show different stress states in the samples. The atomic arrangement is more actively in strain localization regions than in the matrix. The dilatation or annihilation of Voronoi volume during plastic deformation can be seen as a result of the competition between ordering via structure relaxation and shear stress-induced deformation.

## Methods

To understand the structure-property relationships in phase separating MGs molecular dynamics (MD) were carried out using the software LAMMPS. The Fe_50_Cu_50_ glass was simulated using the embedded atom method (EAM) developed by Bonny *et al*.^37^. The slab samples with a composition of Fe_50_Cu_50_ in dimensions of about 43.5 nm(*X*) × 22.1 nm(*Y*) × 2.9 nm(*Z*) contained 230400 atoms was produced by quenching it from the melt. The system was firstly fully equilibrated at 3000 K and zero external pressure for 1ns in a NPT ensemble using a Nose-Hoover thermostat to ensure chemical homogeneity. For all simulations a constant integration time step of 1 fs was used. Periodic boundary conditions (PBCs) were initially applied in all three directions. The Fe_50_Cu_50_ liquid shows a strong interaction between homogeneous atom pairs at a temperature of 1873 K^38^. In order to create alloys with different degree of phase segregation, the system was instantly relaxed at 1800 K for 0, 1, 2, 5 and 10 ns, respectively (being named S1, S2, S3, S4 and S5, respectively). Then the samples were cooled to 100 K at a rate of 10^13^ K • s^−1^ in order to form MGs. Afterwards, PBCs were imposed in the *X* and *Z* directions, while free surfaces were used in the *Y* direction to allow shear offset on free surfaces to take place^18^. Before X direction compressive loading, the samples were first allowed to relax for another 1ns. The Z direction was kept at zero external pressure during compression. The behavior of materials can be significantly different with different strain rates^36^. The effects of different strain rates are beyond the scope of present study, and hence we focus our discussions on the same strain rate. All samples were deformed by applying a constant strain rate of 10^8^ s^−1^ (quasi-static compression) at a constant temperature of 100 K.

The deformation process was monitored by the atomic local shear strain measured by 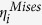, defined by Shimizu *et al*.^39^, which has been incorporated into AtomEye^40^. The local shear strains were referenced to the relaxed samples before compression. The topological SRO and Voronoi volume of clusters were analyzed using the Voronoi tessellation method^19^, which divides the simulation cell into Voronoi polyhedra around each atom. Coordination numbers can be also determined by this method for atoms sharing a common cell surface are considered nearest neighbors. The polyhedron around each atom can be characterized by the Voronoi index (*n*_*3*_, *n*_*4*_, *n*_*5*_, *n*_*6*_), where *n*_*i*_ denotes the number of *i*-edged faces of the Voronoi polyhedron. For studying the chemical SRO, we have calculated the nearest neighbor correlation index *C*_*ij*_ (*i, j* = Fe, Cu)^21^,^25^.

## Additional Information

**How to cite this article**: Peng, C.-X. *et al*. Correlation between structural heterogeneity and plastic deformation for phase separating FeCu metallic glasses. *Sci. Rep.* **6**, 34340; doi: 10.1038/srep34340 (2016).
